# Supplementary material for: Long-term muscle-specific overexpression of DOK7 in mice using AAV9-tMCK-DOK7
Source: Mol Ther Nucleic Acids. 2023 Aug 2;33:617–28. doi: 10.1016/j.omtn.2023.07.036 (PMC10457688; doi:10.1016/j.omtn.2023.07.036)
Supplement: Document S1. Figures S1 and S2 and Tables S1–S3 [file mmc1.pdf]

OMTN, Volume *33*

Supplemental information

Long-term muscle-specific overexpression of DOK7 in mice using AAV9-tMCK-DOK7

Yu-Ting Huang, Hannah R. Crick, Helena Chaytow, Dinja van der Hoorn, Abrar Alhindi, Ross A. Jones, Ralph D. Hector, Stuart R. Cobb, and Thomas H. Gillingwater

Table S1. Histopathology incidence in 3-month-old animals

Tissue	Sex		Low Dose	High Dose	Untreated	Finding incidence	Finding Remark
Brain	M	No Examined	1	1	1	-	
		No. Unremarkable	1	1	1	0	
	F	No Examined	2	2	2	-	
		No. Unremarkable	2	2	2	0	
Diaphragm	M	No Examined	1	1	1	-	
		No. Unremarkable	1	1	1	0	
	F	No Examined	2	2	2	-	
		No. Unremarkable	2	2	2	0	
Heart	M	No Examined	1	1	1	-	
		No. Unremarkable	1	1	1	0	
	F	No Examined	2	2	2	-	
		No. Unremarkable	2	2	2	0	
Kidney	M	No Examined	1	1	1	-	
		No. Unremarkable	1	1	1	0	
	F	No Examined	2	2	2	-	
		No. Unremarkable	1	2	2	1	Hypertrophy
Liver	M	No Examined	1	1	1	-	
		No. Unremarkable	1	1	0	1	Mixed cell infiltration
	F	No Examined	2	2	2	-	
		No. Unremarkable	2	1	0	3	Mixed cell infiltration
Lungs	M	No Examined	1	1	1	-	
		No. Unremarkable	1	1	1	0	
	F	No Examined	2	2	2	-	
		No. Unremarkable	2	2	2	0	
Soleus	M	No Examined	1	1	1	-	
		No. Unremarkable	1	1	1	0	
	F	No Examined	2	2	2	-	
		No. Unremarkable	2	2	2	0	
Spleen	M	No Examined	1	1	1	-	

		No. Unremarkable	0	0	0	3	Extramedullary Hematopoiesis
	F	No Examined	2	2	2	-	
		No. Unremarkable	0	0	0	3	Extramedullary Hematopoiesis
Testes	M	No Examined	1	1	1	-	
		No. Unremarkable	1	1	1	0	

Table S2. Histopathology incidence in 6-month animals

Tissue	Sex		Low Dose	High Dose	Untreated	Finding incidence	Finding Remark
Brain	M	No Examined	2	2	2	-	
		No. Unremarkable	2	2	2	0	
	F	No Examined	1	1	1	-	
		No. Unremarkable	1	1	1	0	
Diaphragm	M	No Examined	2	2	2	-	
		No. Unremarkable	2	1	2	1	Myofibre degeneration
	F	No Examined	1	1	1	-	
		No. Unremarkable	1	1	1	0	
Heart	M	No Examined	2	2	2	-	
		No. Unremarkable	2	2	2	0	
	F	No Examined	1	1	1	-	
		No. Unremarkable	1	0	1	1	Mononuclear cell infiltration
Kidney	M	No Examined	2	2	2	-	
		No. Unremarkable	1	2	2	1	Tubular basophilia
	F	No Examined	1	1	1	-	
		No. Unremarkable	1	1	1	0	
Liver	M	No Examined	2	2	2	-	
		No. Unremarkable	1	1	2	2	Mixed cell infiltration
	F	No Examined	1	1	1	-	
		No. Unremarkable	0	1	0	2	Mixed cell infiltration
Lungs	M	No Examined	2	2	2	-	
		No. Unremarkable	2	2	2	0	
	F	No Examined	1	1	1	-	

		No. Unremarkable	1	1	1	0	
Soleus	M	No Examined	2	2	2	-	
		No. Unremarkable	2	0	2	2	Myofibre degeneration
	F	No Examined	1	1	1	-	
		No. Unremarkable	0	1	0	2	Myofibre degeneration
			0	0	1	2	Myofibre regeneration
Spleen	M	No Examined	2	2	2	-	
		No. Unremarkable	0	0	0	6	Extramedullary Hematopoiesis
	F	No Examined	1	1	1	-	
		No. Unremarkable	0	0	0	3	Extramedullary Hematopoiesis
Testes	M	No Examined	2	2	2	-	
		No. Unremarkable	2	2	2	0	
Ovary	F	No Examined	1	1	1	-	
		No. Unremarkable	1	1	1	0	

Table S3. Incidence and severity of select Jasmone-related microscopic findings in the soleus muscle at 6 months

		Males			Females		
	Treatment	Untreated	Low Dose	High Dose	Untreated	Low Dose	High Dose
	No Animals	3	3	3	3	3	3
Myofibre Degeneration	Minimal	0	0	2	0	1	0
	Mild	0	0	0	1	0	0
Myofibre Regeneration	Minimal	0	0	0	0	1	1

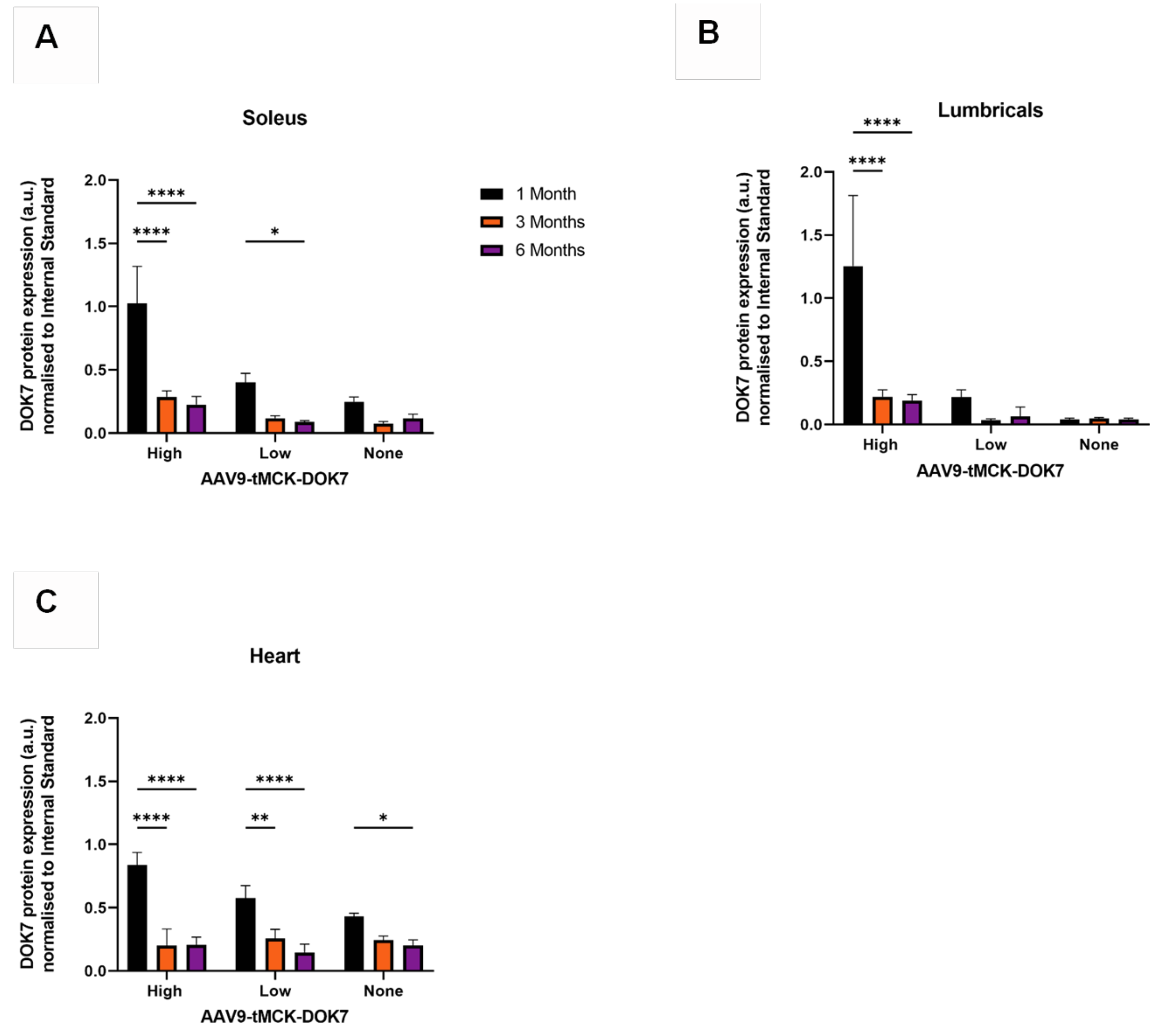

Figure S1. Changes in DOK7 expression over 6 months following AAV9-tMCK-DOK7 treatment

(A) In the soleus muscle DOK7 expression significantly decreased between 1 month of age/post-injection and 3 months following high-dose treatment, and between 1 and 6 months following high and low dose treatments. (B) In the hindlimb lumbrical muscles DOK7 protein expression was significantly decreased after 1-month following injection of the hihg dose only. (C) There was a decrease in endogenous DOK7 levels between 1 and 6-months of age in the heart in untreated animals, as well as a decrease following high and low dose treatment. Data are presented as means +/- SEM. One-way ANOVAs with Sidak's multiple comparisons. **** $p < 0.0001$, ** $p < 0.01$, * $p < 0.05$.

A

B

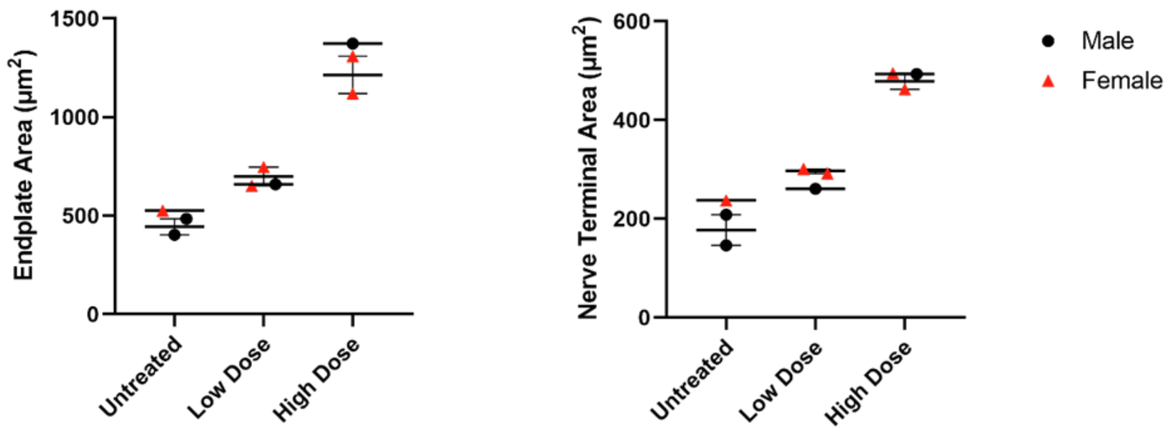

Figure S2. Sex does not impact NMJ parameters in 6-month lumbrical muscles.

Data points produced from female mice are highlighted as red triangles, and those from male mice in black circles. In endplate area (A) and nerve terminal area (B) there is no evidence that sex affects NMJ size in response to AAV9-tMCK-DOK7 treatment at a low or high dose, nor a natural distinction between untreated male and female mice.
