# Supplementary material for: Long-term muscle-specific overexpression of DOK7 in mice using AAV9-tMCK-DOK7
Source: Mol Ther Nucleic Acids. 2023 Aug 2;33:617–28. doi: 10.1016/j.omtn.2023.07.036 (PMC10457688; doi:10.1016/j.omtn.2023.07.036)
Supplement: Document S2. Article plus supplemental information [file mmc2.pdf]

Molecular Therapy
Nucleic Acids
Original Article

Long-term muscle-specific overexpression of DOK7 in mice using AAV9-tMCK-DOK7

Yu-Ting Huang,[1,2,5] Hannah R. Crick,[1,2,5] Helena Chaytow,[1,2] Dinja van der Hoorn,[1,2] Abrar Alhindi,[1,2,3] Ross A. Jones,[1,2] Ralph D. Hector,[4] Stuart R. Cobb,[4] and Thomas H. Gillingwater[1,2]

[1]Edinburgh Medical School: Biomedical Sciences, University of Edinburgh, Edinburgh EH16 4SB, UK; [2]Euan MacDonald Centre for Motor Neuron Disease Research, Edinburgh EH16 4SB, UK; [3]Faculty of Medicine, Department of Anatomy, King Abdulaziz University, Jeddah 21589, Saudi Arabia; [4]Neurogene Inc, 535 W 24th St, New York, NY 10011, USA

Neuromuscular junction (NMJ) dysfunction underlies several diseases, including congenital myasthenic syndromes (CMSs) and motor neuron disease (MND). Molecular pathways governing NMJ stability are therefore of interest from both biological and therapeutic perspectives. Muscle-specific kinase (MuSK) is necessary for the formation and maintenance of post-synaptic elements of the NMJ, and downstream of tyrosine kinases 7 (DOK7) is crucial for activation of the MuSK pathway. Overexpression of DOK7 using AAV9 has been shown to ameliorate neuromuscular pathology in pre-clinical disease models of CMS and MND. However, long-term consequences of DOK7 expression have been sparsely investigated and targeted overexpression of DOK7 in skeletal muscle yet to be established. Here, we developed and characterized a novel AAV9-DOK7 facilitating forced expression of DOK7 under a skeletal muscle-specific promoter. AAV9-tMCK-DOK7 was systemically delivered to newborn mice that were monitored over 6 months. DOK7 overexpression was restricted to skeletal muscles. Body weight, blood biochemistry, and histopathological assessments were unaffected by AAV9-tMCK-DOK7 treatment. In contrast, forced expression of DOK7 resulted in enlargement of both the pre- and post-synaptic components of the NMJ, without causing denervation. We conclude that muscle-specific DOK7 overexpression can be achieved in a safe manner, with the capacity to target NMJs *in vivo*.

INTRODUCTION

The neuromuscular junction (NMJ) is a critical site of synaptic connection between a skeletal muscle fiber and its innervating lower motor neuron, necessary for all movements of the body.[1,2] Each functional NMJ consists of three cellular parts: a pre-synaptic motor nerve terminal that releases the neurotransmitter acetylcholine (ACh), a post-synaptic motor endplate rich in acetylcholine receptors (AChRs), and one or more non-myelinating, terminal Schwann cells.[3] Uncovering the mechanisms that regulate the formation, stability, aging, and pathology of NMJs is of critical importance for our understanding of development and disease in the neuromuscular system.[4–6]

Prior to nerve contact and innervation, AChRs cluster in the central region of myotubes in a process known as muscle pre-patterning. Muscle pre-patterning is a motor neuron-independent, but muscle-specific kinase (MuSK)-dependent process. Upon innervation, Agrin released from motor nerve terminals interacts with its receptor, low-density lipoprotein receptor-related protein 4 (Lrp4), which associates with MuSK to fine-tune neuromuscular synaptogenesis and the patterning of motor endplates.[7] MuSK is also required for the maintenance of mature NMJs, with inactivation of MuSK causing disassembly of NMJs, motor defects, and weight loss in adult rodents.[8–10]

For a long time, it was believed that the Agrin-Lrp4-MuSK pathway was the sole trigger for neuromuscular synaptogenesis. However, AChR clustering at the central region of motor endplates starts prior to innervation by a motor neuron, and the domain for phosphotyrosine binding (PTB) located in the juxtamembrane region of MuSK is essential for AChR aggregation, implying the presence of an internal activator of the MuSK pathway.[11] Downstream of tyrosine kinases 7 (DOK7), comprising a PTB and a pleckstrin-homology domain in its N terminus and a Src homology (SH2) domain in its C terminus, was subsequently identified as a direct binding partner of MuSK.[12–15] Bergamin et al. and Okada et al. proposed that DOK7 activates MuSK for muscle pre-patterning. Indeed, *Dok7*-deficient mouse embryos failed to form AChR clusters at embryonic days (E) 14.5 and E18.5 and died soon after birth.[12,13] Then, upon innervation, ACh is widely released from pre-synaptic vesicles and diffuses into the synaptic cleft. AChR clusters are subsequently maintained in the locality of nerve terminals by the combined action of DOK7 and nerve-derived agrin, which activates MuSK, while AChRs that are peripheral to the nerve terminal are eliminated.[12]

Genetic studies have revealed that mutations in the SH2 domain of *DOK7* contribute to a subgroup of congenital myasthenic syndromes (DOK7-CMS), an inherited neuromuscular disorder characterized by fatigable muscle weakness.[16,17] The most common

Received 4 April 2023; accepted 31 July 2023;
https://doi.org/10.1016/j.omtn.2023.07.036.

[5]These authors contributed equally

Correspondence: Thomas H. Gillingwater, Edinburgh Medical School: Biomedical Sciences, University of Edinburgh, Edinburgh EH16 4SB, UK.
E-mail: t.gillingwater@ed.ac.uk

This is an open access article under the CC BY license (http://creativecommons.org/licenses/by/4.0/).

mutation in DOK7-CMS is an exon 7 frameshift duplication (*c.1124_1127dupTGCC*), leading to the loss of two tyrosine residues and truncated DOK7 in the C-terminal region.[18] The C-terminal region of DOK7 has previously been shown to be important but not essential for MuSK activation, while overexpression of C terminus truncated DOK7 rescued *Dok7*-deficient mice from neonatal lethality.[19,20] In addition, loss-of-function analysis of the two tyrosine residues (e.g., tyrosine to phenylalanine mutation) revealed reduced MuSK activity and AChR clustering in cultured myotubes,[20] but mice carrying this mutation appeared relatively healthy and fertile until adulthood.[21] The majority of human DOK7-CMS patients carrying the *c.1124_1127dupTGCC* mutation live to adulthood, while mice carrying the same mutation suffer premature death.[16,19,21–23] Oury et al. reported that a mixed genetic background DOK7-CMS mouse has a significantly extended lifespan compared to pure inbred mice carrying the same mutation. This suggests that unknown modifiers may impact disease severity and that the same mechanism may contribute, at least in part, to symptoms observed in human patients.

The importance of NMJ stability has been extensively documented across a wide range of conditions, including aging, myasthenia gravis, congenital myasthenic syndromes, sarcopenia, and the motor neuron diseases spinal muscular atrophy (SMA) and amyotrophic lateral sclerosis (ALS).[12,24–28] As a result, targeting of the agrin-Lrp4-MuSK-DOK7 pathway represents an obvious potential therapeutic candidate for stabilizing the NMJ, and it has been explored in several pre-clinical studies. For example, genetic muscle-specific restoration of DOK7 rescued motor function and improved survival in DOK7 myasthenia mice.[22] Similarly, adeno-associated virus (AAV)-driven overexpression of DOK7 ameliorated neuromuscular symptoms in DOK7-CMS mice[22] and in mouse models of ALS[29] and SMA.[30] Furthermore, recent research showed that DOK7 overexpression improves NMJ regeneration in both extensor digitorum longus and tibialis anterior muscles after sciatic nerve injury.[31]

Despite impressive benefits being described for AAV9-DOK7 treatment across a range of disease contexts, to date, overexpression has only been achieved under the control of a ubiquitous expression cytomegalovirus (CMV) promoter. For example, in one representative prior study, such an AAV9 vector was used to overexpress DOK7 in a model of DOK7 myasthenia, with mice reported to survive more than 1 year with no apparent abnormality.[22] Besides this example, however, most studies have only examined the benefits and consequences of DOK7 overexpression over a relatively short time frame (3 months maximum), meaning that long-term safety data is currently lacking, representing a potential hurdle for clinical application.

We have designed and assessed a novel AAV serotype 9 vector forcing expression of human DOK7 in a muscle-specific manner under the muscle creatine kinase (MCK) promoter: AAV9-tMCK-DOK7. In healthy, wild-type mice, DOK7 was robustly upregulated 1 month post administration of AAV9-tMCK-DOK7 in multiple skeletal muscles, without leakage of expression across other major organs and tissues. Long-term expression at 6 months post administration resulted in a dose-dependent enlargement of NMJs without evoking changes in innervation patterns, body weight, overall organ pathology, or blood biochemistry measurements, demonstrating that forced expression of muscle-specific DOK7 is both effective and well-tolerated *in vivo*.

RESULTS

Muscle-specific expression of DOK7 in mice using AAV9-tMCK-DOK7

Initial validation of tissue-specific DOK7 overexpression following treatment with a newly constructed AAV9-tMCK-DOK7 viral vector (see materials and methods for details) was carried out in wild-type neonatal mice. Intravenous administration of either 5e11 vector genome (vg, high dose) or 1.25e11 vg (low dose) per mouse was performed on the day of birth. Expression of human DOK7 protein was driven by the muscle-targeted promoter MCK (Figure 1A). Protein expression was confirmed in selected skeletal muscles and organs in 1-month-old mice using quantitative fluorescent western blot (Figure 1B). As this approach analyzes the expression of both human and mouse DOK7 expression, without distinguishing between the two, it remains possible that endogenous levels of mouse DOK7 might be affected after forced expression of human DOK7. Thus, all subsequent DOK7 expression comparisons reflect a measure of total DOK7 levels from both species in the AAV9-tMCK-DOK7 treated groups, and we will use “overexpression” to describe an increase in total DOK7 levels.

The internal standard used here consisted of a mixture of gastrocnemius muscle protein lysates from both high- and low-dosed mice, allowing us to directly compare DOK7 expression across tissues/organs examined (see materials and methods for details). Endogenous DOK7 levels (without AAV9-tMCK-DOK7 treatment) are shown in Figure 1C, confirming that higher levels of DOK7 were present in skeletal muscle, heart, and spinal cord compared to other tissues and organs. A dose-dependent overexpression of DOK7 was observed in all examined muscles following AAV9-tMCK-DOK7 treatment, without leakage to other organs, demonstrating the specificity of the muscle-specific promoter (Figure 1D).

Next, long-term forced expression of AAV9-tMCK-DOK7 was examined in wild-type mice treated on the day of birth at 3 and 6 months post administration. The hind-paw lumbrical and soleus muscles were chosen for investigation, representing fast- and slow-twitch skeletal muscles respectively. Both muscles showed a significant upregulation of DOK7 protein levels when compared to untreated tissue at both time points following high-dose treatment with AAV9-tMCK-DOK7, with this increased expression notably absent from the heart (Figure 2). Despite a decrease in DOK7 expression from 1 to 3 months following injection (Figure S1), the lumbricals yet presented over a 4-fold increase in DOK7 levels at 3 and 6 months post injection, while in the soleus muscle, an initial 4-fold increase in DOK7 levels at 3 months reduced to

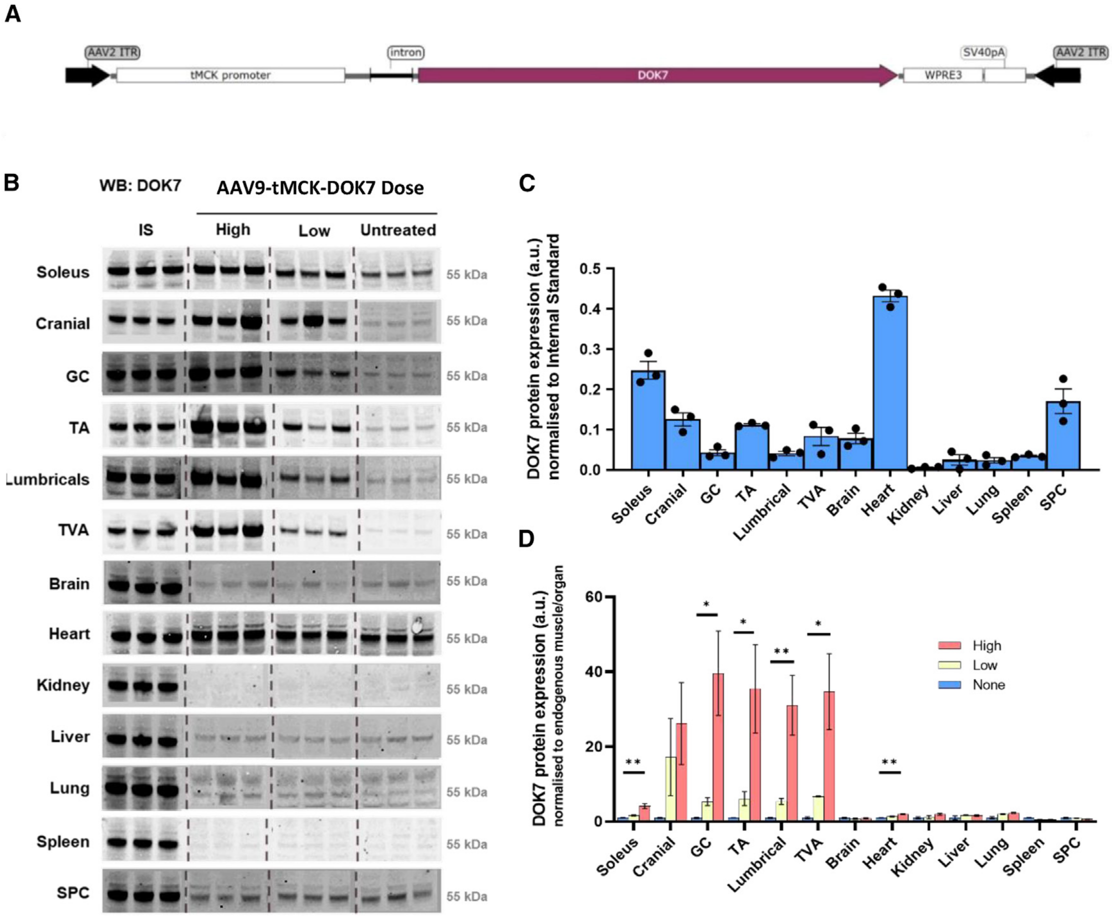

Figure 1. AAV9-tMCK-DOK7 increases DOK7 expression in skeletal muscle

(A) Schematic of the AAV9-tMCK-DOK7 plasmid showing the skeletal muscle targeting tMCK promoter and DOK7 transgene.

(B–D) DOK7 expression across a range of muscles and organs from 1-month-old mice treated with high dose (5e11 vg), low dose (1.25e11 vg), or untreated with AAV9-tMCK-DOK7. "IS" denotes internal standards run in triplicate used to allow comparison between blots. (B) Western blots where the molecular weight of DOK7 is indicated. Dotted lines illustrate where blot sections have been cropped together. (C) Bar graph of endogenous expression in skeletal muscle and tissue shown by quantified western blots. Values are normalized to the average of three DOK7 internal standards (ISs). Each dot represents a single mouse, and bars represent the mean (with SEM) (n = 3 per bar). (D) DOK7 expression in response to high-dose or low-dose treatments were compared by one-way ANOVA with Holm-Šídák's multiple comparisons. Each bar represents an average of three animals (with SEM), normalized to internal standard (IS) and then normalized to endogenous protein levels for that tissue. Expression increased by 40x over endogenous levels in the GC ($p = 0.0114$), and the TA ($p = 0.0242$), hindlimb lumbricals ($p = 0.0074$), and TVA ($p = 0.013$) each showed a marked rise in DOK7. The soleus, which exhibited the highest endogenous levels of DOK7 in skeletal muscle, still expressed ~4x as much DOK7 following high-dose treatment ($p = 0.0032$). There was also a small but significant increase in DOK7 expression in the heart following high-dose AAV9-tMCK-DOK7 treatment ($p = 0.0018$, n = 3 in all groups, one-way ANOVAs with Holm-Šídák's comparisons). SPC, spinal cord; TVA, transverse abdominis; TA, tibialis anterior; GC, gastrocnemius; *$p < 0.05$, **$p < 0.01$.

approximately 2-fold at the 6-month time point. Furthermore, this relative decrease in detectable DOK7 between 1 and 3 months could be attributed to the distribution of a stable number of non-replicating viral vectors through myofibers that grow in size and number during early postnatal life.[32] This finding confirms that DOK7 protein levels can be elevated over a prolonged period in skeletal muscle following a single dose of AAV9-tMCK-DOK7 and also demonstrates the restriction of expression to skeletal muscle.

Given that DOK7 levels were observed to be increased across the long term in mice treated with a high dose of AAV9-tMCK-DOK7 compared to untreated animals, we next wanted to establish whether this had any consequences for the health status of the mice, with a particular focus on any emerging phenotypes in the neuromuscular system. The righting reflex was first used to assess the development of motor function in neonatal pups either untreated or having received a high or low dose of AAV9-tMCK-DOK7. No differences in righting reflex were observed between the three treatment groups (Figure 3A). Gender combined body weight was also indistinguishable between treatment groups at all time points examined from birth (Figure 3B, n = 25, 20, and 10 for each group at 1, 3, and 6 months, respectively).

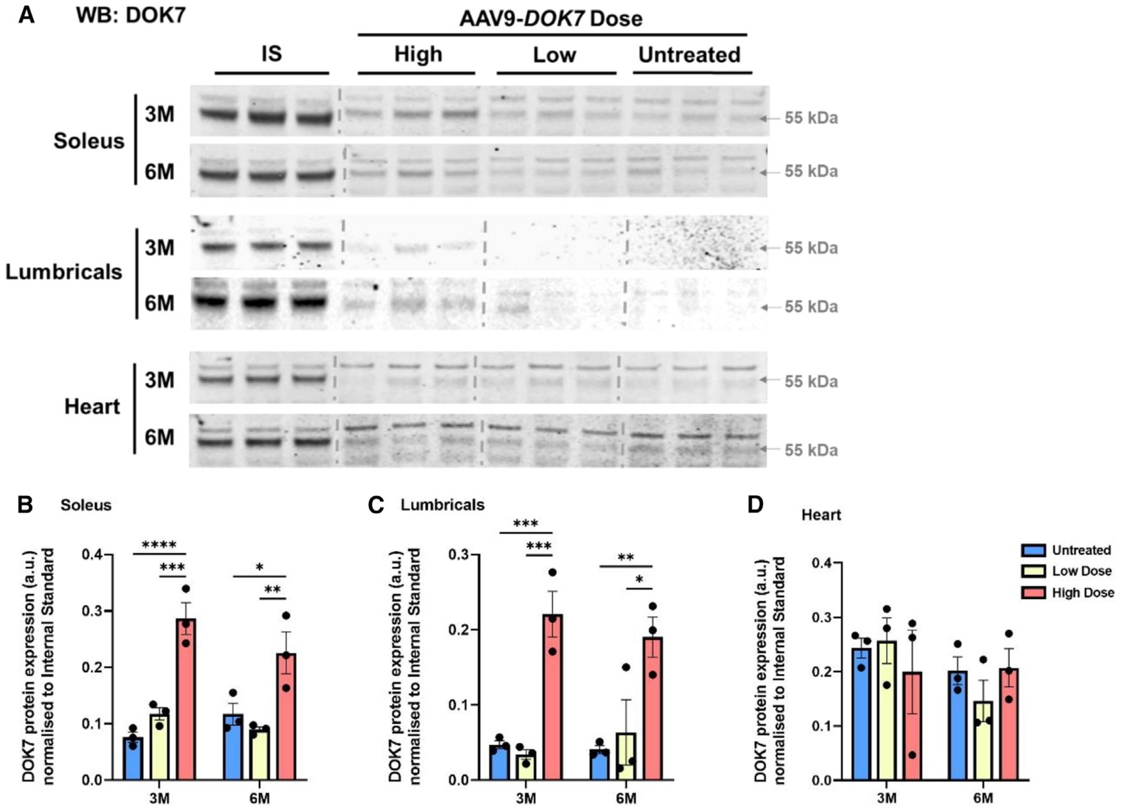

Figure 2. hDOK7 overexpression stimulates long-term elevation of DOK7 protein expression in mouse hindlimb muscles but not the heart

(A) Western blots of tissue lysates probed with anti-DOK7 antibody shown at 55 kDa (middle band). Tissue from three mice each untreated or injected with high- or low-dose AAV9-tMCK-DOK7 was run alongside internal standards (ISs) produced in triplicate, enabling cross-gel comparison. Tissues were taken from mice at 3 months (M) and 6 months of age.

(B–D) Bar charts comparing the effect of dose on tissue-specific DOK7 expression. Western blot data were quantified and normalized to the average value of three internal standards. DOK7 expression was significantly increased at 3 and 6 months following high-dose treatment in the hindlimb soleus muscle (B) and lumbricals (C) but not in heart tissue (D), when compared to untreated controls. Specifically, there was a 3.8-fold increase in DOK7 expression in the soleus 3 months following treatment and a 1.9-fold increase at 6 months (B; $p < 0.0001$ and $p = 0.019$), while in the lumbricals, there was a 4.7-fold increase in DOK7 expression at 3 months and a 4.6-fold increase at 6 months (C; $p = 0.0009$ and $p = 0.0031$) (one-way ANOVAs with Šídák's multiple comparisons, means of n = 3 per group [with SEM], and each data point represents one animal). *$p < 0.05$, **$p < 0.01$, ***$p < 0.001$, ****$p < 0.0001$.

Plasma was taken from mice at both 3- and 6-month time points in order to assay systemic biochemistry. The levels of total protein, albumin, globulin, albumin/globulin ratio, and alanine transaminase were unchanged at all time points examined in the AAV9-tMCK-DOK7-treated mice compared to untreated mice. There were also no differences in the levels of creatinine or aspartate transaminase (AST) at 3 months of age. Due to the limited availability of tissue, creatinine and AST levels were only available from two mice at the 6-month time point (Figures 3C–3I).

Hematoxylin and eosin (H&E) staining was performed on musculoskeletal tissues (soleus and diaphragm) as well as a range of organs including whole brain, heart, lung, liver, spleen, ovary/testes, and kidney from mice taken at both 3 and 6 months of age, with and without AAV9-tMCK-DOK7 treatment. There were no distinguishable differences between any organs examined in 3- and 6-month-old groups nor between muscles at 3-month-old groups. Minimal evidence of myofiber degeneration was present in two soleus samples from both low- and high-dosed groups and in one diaphragm sample from the high-dose group at the 6-month time point. Mild morphological signs of regeneration were present in two of the soleus muscles from the untreated group at the same time point. Minimal myofiber regeneration was found in one mouse from both low- and high-dosed groups at 6 months following AAV9-tMCK-DOK7 treatment (Figure 4 and Table S1).

Taken together, these data confirm the ability to generate prolonged, high expression of DOK7 restricted to skeletal muscle *in vivo* by using a muscle-specific promoter and AAV9-driven delivery. Moreover, sustained expression of DOK7 in skeletal muscle had no observable adverse effects on systemic or neuromuscular health in mice.

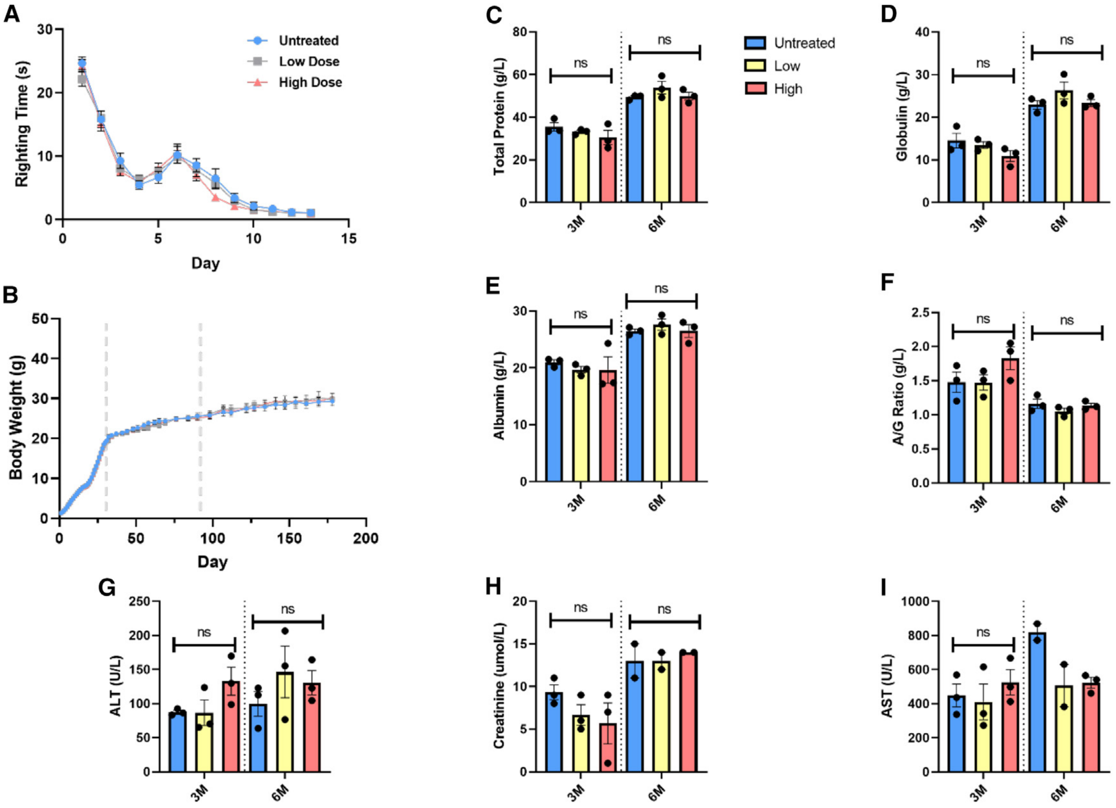

Figure 3. Overexpression of DOK7 in skeletal muscle does not affect animal wellbeing nor motor development

(A) Righting reflex measured from P1 to P13 when all animals consistently achieved the minimum time to right (1 s). Each point represents the average time of approximately 30 animals per group (mean [SEM]). Comparing between AAV9-tMCK-DOK7 doses revealed no difference among high-dose or low-dose treated or untreated animals (ns, n = 88; two-way ANOVA with Tukey's post hoc comparisons).

(B) Body weights (g) of mice from day 1 (day of birth) to 6 months (experimental endpoint). Dotted lines indicate where at day 30 and day 90, mice from each group were sacrificed for tissue, making n per group = 30, 20, and 10 from day 0, day 30, and day 90, respectively (mean and SEM). There was no difference observed between AAV9-tMCK-DOK7 doses (two-way ANOVA with Tukey's multiple comparisons).

(C–I) Plasma biochemistry measuring biomarkers from high- or low-dose-treated animals or untreated controls at 3 or 6 months old. Each data point represents a readout from one mouse, and bars represent averages from three mice (with SEM, one-way ANOVAs). ALT, alanine transaminase; AST, aspartate transaminase; ns, not significant.

Long-term overexpression of DOK7 modifies NMJ morphology without affecting innervation

Next, we examined the impact of forcing expression of DOK7 over the long term on NMJs *in vivo*. Initial investigations (see above) demonstrated that lumbrical muscles from wild-type mice treated with high-dose AAV9-tMCK-DOK7 showed significant overexpression of DOK7 at 1 month (Figure 1D) and 3 and 6 months (Figure 2C). Inspection of lumbrical muscles also revealed relatively low endogenous levels of DOK7 (Figure 1C), making them suitable targets to study the effects of DOK7 overexpression on NMJs.

Figures 5A and 5B show representative confocal micrographs of NMJs from lumbrical muscles at 3 and 6 months after AAV9-tMCK-DOK7 treatment, with α-bungarotoxin labeling AChR endplates and anti-2H3 and anti-SV2 antibodies labeling axons and synaptic vesicles at nerve terminals, respectively. The level of innervation was determined by experimenters in a double-blinded manner (see materials and methods for details). The numbers of healthy NMJs, characterized by full innervation from a single motor axon, and pathological NMJs, characterized by a combination of partial innervation or full denervation, are shown in Figures 5C–5F, revealing no significant change in innervation patterns between the three treatment groups at either time point. This is despite the muscles expressing more than twice the normal physiological levels of DOK7 (Figures 2B and 2C). Thus, long-term overexpression of DOK7 does not lead to any overt neuromuscular pathology *in vivo*.

Given that DOK7 is thought to be capable of modulating the morphological size of NMJs,[22] we next wanted to investigate the impact of long-term overexpression on NMJ morphology. We used the open-source Fiji plugin NMJ morph to analyze multiple morphological parameters of NMJs.[33,34] In the lumbrical muscles from high-dosed AAV9-tMCK-DOK7 mice, AChR endplates and pre-synaptic

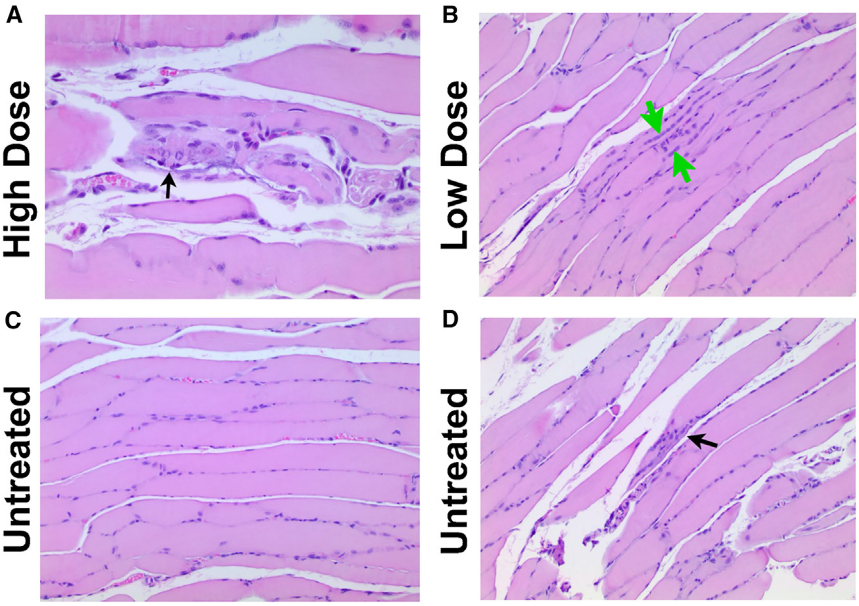

Figure 4. Overexpression of DOK7 in skeletal muscle does not damage myofibers

(A) Micrographs of H&E-stained soleus muscles from 6-month-old animals showing (A) minimal myofiber degeneration in a male treated with high-dose smAAV9-*DOK7* highlighted with a black arrow, 40x.

(B) Skeletal muscle regeneration, characterized by rows of plump nuclei in the central myofiber, in the soleus of a low-dose-treated animal, highlighted by green arrows, 20x.

(C) Normal presentation in an untreated male, 20x.

(D) Focal myofiber degeneration highlighted by the black arrow in the soleus of an untreated animal. For further histology results, see Tables S1–S3.

terminals were both enlarged by approximately 150% at 6 months compared to the untreated group (p = 0.0006 and p = 0.0007 respectively, n = 3 per group, unpaired t test; Figures 6H and 6L), without any concomitant changes in axon diameter (Figure 6D). There was also a modest but significant increase in the AChR endplate and nerve terminal areas of NMJs from low-dosed mice at 6 months post injection (Figures 6F and 6J). These changes appear to be a consequence of longer-term DOK7 overexpression, as there were no changes in either AChR endplate or nerve terminal areas in AAV9-tMCK-DOK7-treated mice 3 months after treatment (Figures 6E, 6G, 6I, and 6K). Moreover, there were no gender differences observed in these NMJ parameters (Figure S2). Taken together, these data reveal that long-term overexpression of DOK7 using AAV9-tMCK-DOK7 is not only systemically safe but also leads to an increase in the overall size of NMJs.

DISCUSSION

The NMJ represents a crucial point of vulnerability across a range of neurological and neuromuscular conditions, where gene therapies are emerging as powerful therapeutic interventions (e.g., Zolgensma for SMA and Tofersen for SOD1-linked ALS patients).[35,36] In this study, we have expanded upon exciting research suggesting that AAV9 vectors driving DOK7 expression have the potential to stabilize the neuromuscular system across a range of disease indications. We developed a skeletal muscle-specific AAV9-tMCK-DOK7 and show that single-dose intravenous delivery in mice leads to long-lasting, dose-dependent increases in DOK7 across a range of skeletal muscles. Overexpressed DOK7 did not alter overall health parameters such as body weight and blood biochemistry readouts nor induce muscle pathology. However, overexpression of DOK7 resulted in enlargement of NMJs in a dose- and time-dependent manner, without affecting the innervation status of muscle.

AAV9-driven DOK7 overexpression has been used in numerous pre-clinical studies, including DOK7-CMS,[21,22] SMA,[30] and ALS.[29] For example, Ueta et al. used systemically expressed AAV9-DOK7 in aged mice at 24 months old, revealing improved motor function without evident abnormalities.[23] Arimura et al. also reported that AAV9-DOK7-treated DOK7-myasthenia mice survived more than 1 year, in comparison to the usual lifespan of 20 days if left untreated.[22] However, most previous studies only explored the potential benefits of DOK7 gene therapy over a short duration (3 months or less) and in animals where overexpression was driven by a ubiquitous promoter (e.g., CMV). These studies illustrate the benefits of increased expression of DOK7; for example, robust and ubiquitous CMV-driven DOK7 expression generated extensive NMJ enlargement in a mouse model of DOK7-CMS, even more so than this study, and treated mice showed body weight and motor function equivalent to wild types after untreated animals had reached endpoints.[22] However, AAV9-CMV-driven target gene expression is highly expressed in major organs, including heart, liver, and lung as well as skeletal muscles.[37] In this study, we therefore took the advantage of AAV9 for its skeletal muscle tropism while avoiding physiologically abnormal expression previously evidenced in non-muscle tissue. Furthermore, long-term overexpression of DOK7 restricted to muscle did not lead to abnormalities in major organs nor blood biochemistry, implying no long-term safety concerns of supraphysiological DOK7 expression. Thus, AAV9-DOK7 with expression restricted to skeletal muscle is likely to represent a safer and more targeted therapeutic for delivery to humans with neuromuscular conditions.

Previously, DOK7 overexpression has been shown to increase NMJ size in a range of mouse models of motor dysfunction. Enlarged motor endplates have been demonstrated in response to AAV9-delivered DOK7 in an Smn2B/– model of SMA, DOK7 myasthenia, and Emery Dreifuss muscular dystrophy.[21,22,30] Furthermore, there is existing evidence of increased pre- and post-synaptic areas following DOK7 overexpression in aged mice and $SOD1^{G93A}$ models of ALS.[23,29] Our evidence shows that an early dose of AAV9-tMCK-DOK7 can have long-lasting effects, with the benefits of the high dose reflected

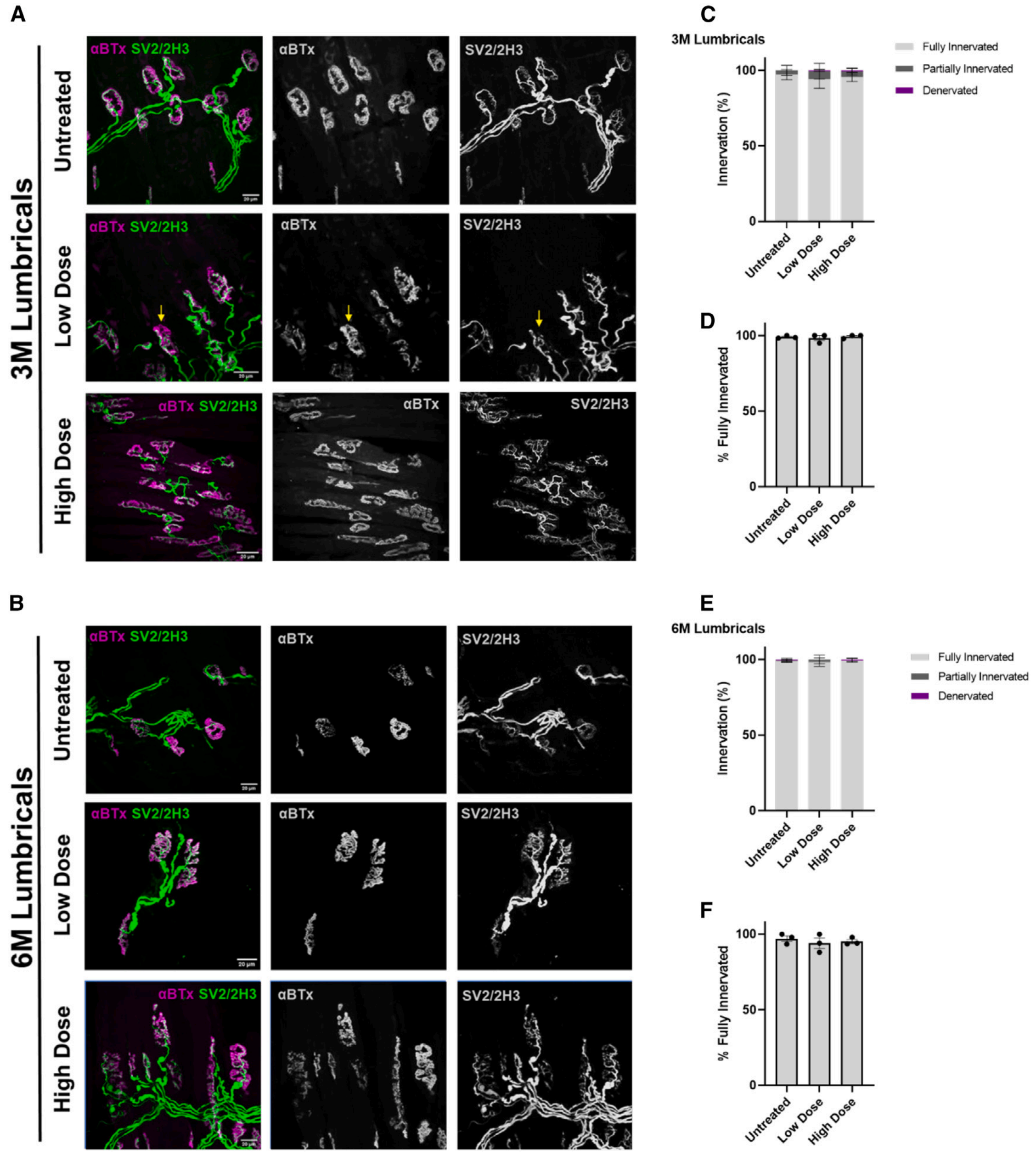

Figure 5. Innervation of hindlimb lumbrical muscle NMJs is not affected by AAV9-tMCK-DOK7 overexpression

(A and B) NMJs labeled with α-bungarotoxin (AChRs) in magenta and anti-synaptic vesicle 2 (SV2) and anti-2H3 (neurofilaments) in green are shown in representative confocal micrographs. High-dose and low-dose AAV9-MCK-DOK7-treated and untreated lumbricals were taken at 3 months (A) and 6 months (B). Yellow arrows indicate partially denervated NMJs. Scale bars (white), 20 μm.

(C and E) Percentages of NMJs fully innervated, partially innervated, or denervated presented by AAV9-tMCK-DOK7 dose condition. Mean percentages were computed from counts from three mice per condition (mean [SEM]). A minimum of 28 NMJs per muscle from each mouse were evaluated in tissue from 3- (C) and 6- (E) month-old mice. (D and F) Bar charts comparing the percentage of fully innervated NMJs between treatment conditions, showing results from the lumbricals taken from mice at 3 (D) and 6 (F) months old. Each point represents data from one animal and each bar represents an average of three animals with SEM. There was no difference between treatments as assessed a Kruskal-Wallis test with Dunn's multiple comparisons (ns) (total number of NMJs analyzed per condition: untreated: 3M lumbricals, n = 232; 6M lumbricals, n = 190; low dose: 3M lumbricals, n = 129; 6M lumbricals, n = 165; high dose: 3M lumbricals, n = 222; 6M lumbricals, n = 134).

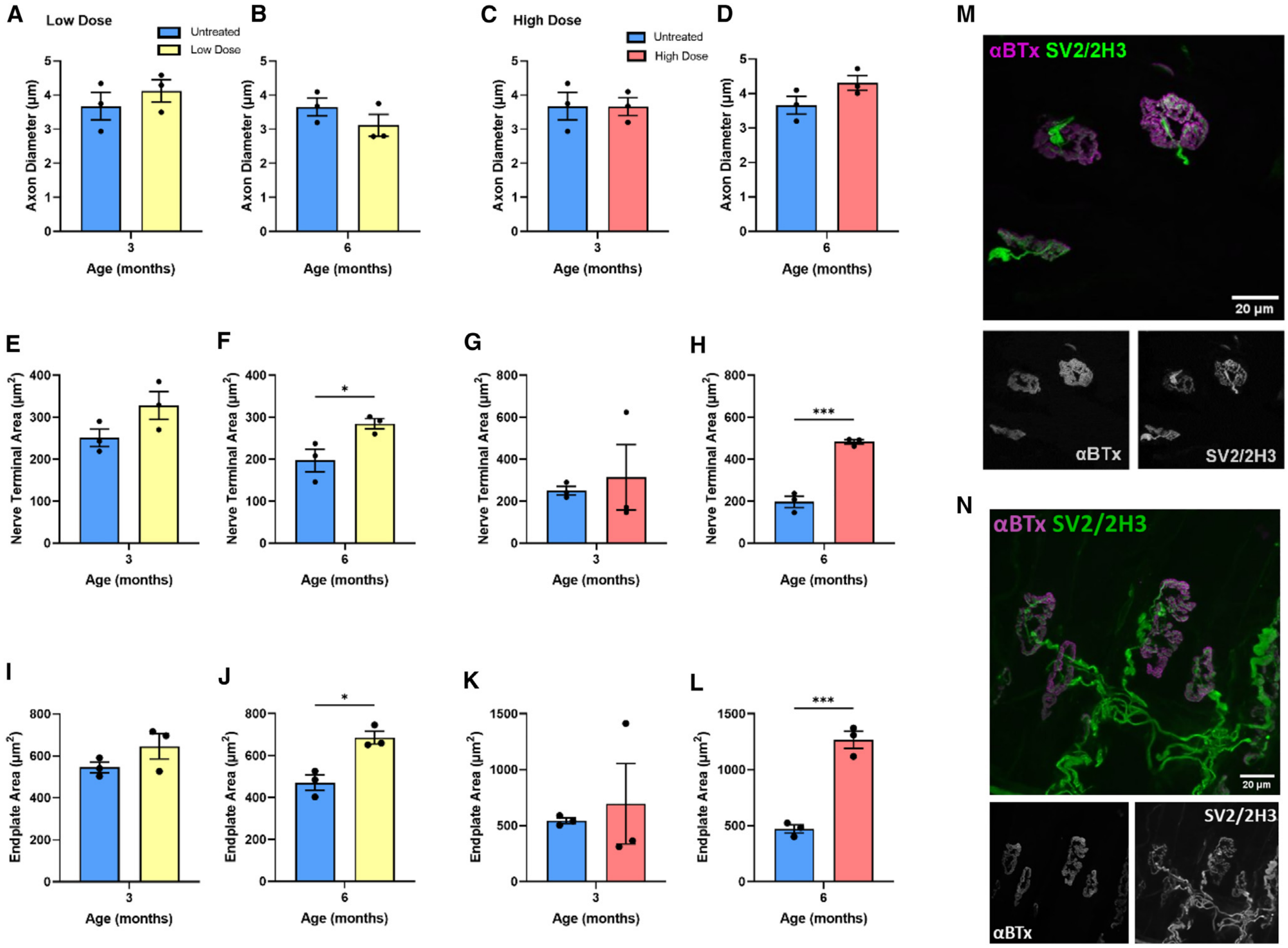

Figure 6. Long-term enlargement of lumbrical NMJs after AAV9-tMCK-DOK7 treatment

(A–L) Bar charts comparing NMJ morphology in mice injected with low- or high-dose AAV9-tMCK-DOK7, 3 or 6 months prior. One data point represents the average of a minimum of 20 NMJs from the hindlimb lumbrical muscles of a single mouse. Each bar represents the mean (SEM) (n = 3 per group). At 6 months post injection, there was a significant increase in endplate area in low- (J; $p = 0.011$) and high-dose (L; $p = 0.0006$) -treated animals, with an over 100% increase following the high dose. Nerve terminal area was also increased 6 months after low- (F; $p = 0.042$) and high-dose (H; $p = 0.0007$) treatment, as assessed using unpaired t tests. Untreated groups were repeatedly used in comparison with low- or high-dosed muscle samples using unpaired t tests for comparisons. (M and N) Confocal micrographs of NMJs labeled with α-bungarotoxin (AChRs) in magenta and anti-synaptic vesicle 2 (SV2) and anti-2H3 (neurofilaments) in green. Images are representative of lumbrical muscles taken from 6-month-old mice treated with high-dose AAV9-tMCK-DOK7 (N) or untreated (M) at P1. *$p < 0.05$, $p^{***} < 0.001$.

in enlargement of NMJs, particularly at 6 months post injection. Smaller, but statistically significant, changes were also observed in low-dose-treated mice even after the overexpression of DOK7 had returned to physiological levels at the 3- and 6- month time points. This builds on previous findings implying that processes that lead to enlargement of the NMJ orchestrated by overexpressed DOK7 are maintained over a sustained period of time. Our work further supports the safety of such treatments as there were no immediate neuromuscular defects, defined by righting reflex in pre-weaning age, nor long-term abnormalities in body weight between groups, indicating that all mice continued to have free access to food and water. Blood biochemistry and histopathology were normal despite the long-term increase in DOK7 and changes to the morphology of the NMJ. With regard to NMJ function, after AAV9-tMCK-DOK7 administration, Ueta et al. reported an increase of maximal amplitude of compound muscle action potentials with enhanced innervation of the muscle and enlarged neuromuscular junction size.[23] Another study using salbutamol, a drug licensed for DOK7-CMS human patients, in a mouse model of DOK7-CMS demonstrated an increased number of active NMJs and percentage of fibers with detected miniature endplate potentials.[38] Both studies therefore showed that targeting DOK7 in mice can modulate neuromuscular synaptic function. Thus, further investigations into the therapeutic effects of muscle-specific overexpressed human DOK7 should aim to incorporate neurophysiological experiments, as well as behavioral tests such as rotarod or grip strength, in order to clarify whether the observed increases in NMJ

size translate to functional modulation. It will also be important to now test whether AAV9-tMCK-DOK7 administration can have similar beneficial therapeutic effects in DOK7-CMS models[22] or other motor neuron disease models such as SMA[39] and ALS.[29,40]

It is interesting to note that we observed high endogenous levels of DOK7 protein in the heart of wild-type mice, in keeping with previous reports.[12] It remains unclear what role(s) DOK7 plays in the heart. One case report associated DOK7 mutations in humans with mitral valve prolapse in siblings with CMS,[41] but similar phenotypes have not been identified in larger-scale studies of DOK7-CMS patients,[17] and no change in heart histology nor detriment to heart function has been reported in mice systemically overexpressing DOK7.[12,22] Thus, it remains unclear why DOK7 is highly expressed in skeletal muscle and in heart but not in other major organs such as brain, lung, and liver in humans.[12] Interestingly, MuSK is not thought to be expressed in the heart nor involved in any neuro-cardiac interactions,[42] suggesting that DOK7 might have additional roles beyond AChR clustering. Based on our experiments in mice, it is not yet possible to rule out longer-term effects of DOK7 overexpression on cardiac function (over years rather than months), and such changes may be exacerbated in patients with existing heart defects. Importantly, therefore, although DOK7 expression in the heart of untreated animals remained high over the time course of our study, we saw no additional increase in cardiac DOK7 levels resulting from AAV9-tMCK-DOK7 treatment at either the 3- or 6-month time point. Thus, the use of skeletal-muscle-restricted AAV9-DOK7 removes any concerns that may arise due to potentially unwanted impacts on cardiac muscle *in vivo*.

In summary, we have demonstrated the long-term safety and efficacy of muscle-specific AAV9-DOK7 gene therapy in mice. Efficient transduction of the transgene resulted in dose-dependent increases in DOK7 levels across a range of skeletal muscles, enhancing NMJ size without inducing any detrimental change in physiological function or neuromuscular health in animals. Thus, muscle-specific AAV9-DOK7 gene therapy represents an attractive therapeutic strategy to develop treatments for patients with a wide range of neuromuscular disorders.

MATERIALS AND METHODS

Animals

Wild-type FVB/N mice purchased from Charles River (Tranent, Scotland) were used throughout this study (incorporating both sexes). All animal procedures and breeding were performed in accordance with UK Home Office guidelines (PPL P92BB9F93) and were approved by an internal ethics committee at the University of Edinburgh.

Generation and delivery of AAV9-tMCK-DOK7

AAV9-DOK7 is a recombinant serotype 9 AAV encoding a wild-type human *DOK7* transgene. DNA fragments containing a triple-tandem muscle creatine kinase (*tMCK*) promoter, a chimeric intron, a human *DOK7* DNA coding sequence (NCBI GeneID: 285489), a shortened woodchuck hepatitis virus posttranscriptional regulatory element, and a simian vacuolating virus 40 polyadenylation signal were synthesized by GENEWIZ (South Plainfield, NJ, USA). Constructs were subsequently cloned into the baculovirus vector V445-ss-pFB by Virovek (Hayward, CA, USA). The resultant vector contained the DOK7 gene therapy construct flanked by AAV2 inverted terminal repeats and a Tn7L recognition sequence to make the vector compatible with baculovirus AAV production. AAV9-DOK7 was generated by Virovek using a baculovirus expression vector system-based process and *Spodoptera frugiperda* (Sf9) insect cells. The final product named AAV9-tMCK-DOK7 was formulated in phosphate-buffered saline (PBS) containing 0.001% Poloxamer 188.

Wild-type FVB/N mice were treated with either a high dose (5e11) or low dose (1.25e11) of AAV9-tMCK-DOK7 vector genomes (vg) via intravenous injection of the facial vein performed on the day of birth (postnatal day 1, P1).[43] The average body weight per mouse used in this study was 1.3 g. Untreated littermate mice were used as controls throughout. Body weight was recorded daily from P1 to P30 and then continually assessed a minimum of once weekly until the end of the experiment. Righting time was measured from P1 to P13.[43]

Tissue sampling

Animals, of both sexes, were euthanized at their respective time points via overdose of inhaled isoflurane, and their organs were immediately removed, frozen in liquid nitrogen, and stored at −80°C. Muscles were dissected out and then fixed in 4% paraformaldehyde for 15 min. Fixed skeletal muscle was kept at 4°C in 0.01% sodium-azide until micro-dissection. Muscles were micro-dissected into thin layers to accommodate staining and imaging, and fat and connective tissue were removed to reduce background staining. Tissue taken for histological staining was preserved in 10% neutral buffered formalin, embedded in paraffin, stained with H&E, and assessed by StageBio (Mount Jackson, Virgina).

Blood collection was performed on the same day as tissue dissection. Sterile P1000 pipette tips were pre-coated with 0.5 M EDTA solution. Under terminal anesthesia, blood was collected via the femoral artery and vein from both sides from mice that had received either high or low dose of AAV9-tMCK-DOK7 or were untreated at 3 or 6 months post injection (n = 3 per group). Blood was collected into 0.5 M EDTA-coated, EDTA-containing tubes (EDTA: blood = 1:10), kept on ice, and centrifuged at 2000 RCF for 10 min at 4°C. Plasma was then collected as supernatant and kept at −80°C until further processing. Hematology analysis was performed using the Standard Tox service by IDEXX BioAnalytics (Berkshire, UK).

NMJ immunohistochemistry on whole-mount muscles

Staining procedures were performed as previously described.[2] In short, skeletal muscles were labeled by submersion in the following solutions, all diluted in 1x PBS unless otherwise specified: 0.1 M glycine for 10 min to reduce tissue autofluorescence; 1x PBS wash for 10 min; 2-h permeabilization in 5% Triton X-100; 30-min blocking in 4% bovine serum albumin and 2% Triton X-100. Primary antibody (in block solution) incubation was then completed over 60 h at

4°C with mouse anti-SV2 (synaptic vesicles; 1:50 dilution; DSHB, Iowa City, Iowa) and mouse anti-2H3 IgG (neurofilaments; 1:50 dilution; DSHB).

Tissues were washed in 1x PBS for 4 × 20 min and then incubated in the secondary antibody, 2 μg/mL AlexaFluor-488-conjugated donkey anti-mouse IgG antibody (Cat: A21202, Thermo Scientific, Waltham, Massachusetts) in 1x PBS diluted 1:500, and left overnight at room temperature. Samples were kept in the dark from this stage to prevent photobleaching. A further 4 x 20-min washes in 1x PBS were followed by 15–30 min (muscle dependent) in 2 μg/mL tetramethylrhodamine isothiocyanate-conjugated α-bungarotoxin (acetylcholine receptors; Cat: BTIU00012, VWR International, Radnor, Pennsylvania). Muscle fibers were mounted in Mowoil on glass slides for imaging.

Confocal imaging

A Nikon A1R FLIM confocal laser scanning microscope was used to image NMJs. A minimum of 20, and a mean of 30, suitable NMJs were captured per muscle using 60x or 40x oil immersion objectives. NMJs that were *en face* or up to 10% oblique were considered suitable for analysis. Images were 16-bit, 512 x 512-pixel frame size, with a 0.5-μm z stack interval. The excitation wavelength for the green channel was 488 nm, and the red (shown as magenta) was 561 nm.

Image analysis

Innervation counts and morphological analyses of confocal micrographs were performed on maximum intensity projections of z stacks using ImageJ. Morphological analysis further required the "NMJ-morph" methodology and the BinaryConnectivity plugin.[33] Innervation counts were performed manually, and the experimenter was blinded to the treatment and genotype during image analysis.

Quantitative western blotting

The experimental procedures and analytical methods are as previously described.[44,45] Briefly, samples were homogenized in RIPA buffer (Radio-Immunoprecipitation Assay; Thermo Scientific) supplemented with 1% protease inhibitor (Cat: 78425, Thermo Scientific). Protein-containing supernatant lysate was retrieved after centrifugation and was then quantified using a BCA assay (Micro BCA kit, Cat: 23235; Thermo Scientific) for protein concentration. Internal protein standard (IS) was prepared from 1-month-old gastrocnemius muscles from both high-dosed and low-dosed mice. IS was prepared in large quantity to allow consistent loadings across multiple gels.

After gel electrophoresis, the protein was transferred to a polyvinylidene fluoride and the membranes was stained and imaged for total protein (Revert 700 Total protein stain, TPS; Li-COR 926–11010).[44,46] Blocking in Intercept PBS blocking buffer (Li-COR 927–70001) preceded 4°C overnight incubation in goat anti-DOK7 primary antibody (Cat: AF6398, R&D)[21,47,48] at 1:1,500 in blocking buffer. On the next day, membranes were washed in PBS and then incubated with the secondary antibody donkey anti-goat (Cat: 92632214, Li-COR 1:5,000) for 1 h at room temperature. Blots were visualized on an Odyssey CLX Infrared Imager (Li-COR).

The relative protein expression of DOK7 was quantified using Image Studio Lite 5.2 (Li-Cor). The intensity of the anti-DOK7 bands at 55 kDa in the 800-nm channel were normalized to their respective normalized total TPS values representing total amount of protein in each loading (lane) in order to reduce loading error. DOK7 protein expression for each membrane was further normalized to the average intensity across triplicate ISs to enable accurate comparison between gels.

Statistical analyses

Data were analyzed using GraphPad Prism software version 9.1 (San Diego, California). Values are presented as means (SEM). Datasets with two groups were compared with t tests. For non-parametric datasets containing three or more groups, Kruskal-Wallis tests with Dunn's post hoc comparisons were used. For sets of parametric data containing three or more groups, analyses of variance (ANOVAs) were used with Tukey's or Sidak's post hoc tests. p values <0.05 were considered significant.

DATA AND CODE AVAILABILITY

Datasets used in the current study are available from the corresponding author upon request.

SUPPLEMENTAL INFORMATION

Supplemental information can be found online at https://doi.org/10.1016/j.omtn.2023.07.036.

ACKNOWLEDGMENTS

The authors would like to thank the animal husbandry staff and veterinary surgeons for their contributions and assistance with the current study. The project was funded by NeuroGene, as well as via project grant funding from the My Name'5 Doddie Foundation (to T.H.G.).

AUTHOR CONTRIBUTIONS

This project was administered by Y.-T.H. and T.H.G. Y.-T.H., H.C., T.H.G., and R.A.J. planned and supervised these experiments. R.D.H. and S.R.C. designed and generated viral construct. Y.-T.H., H.R.C., H.C., D.v.d.H., and A.A. conducted laboratory experiments. Y.-T.H., H.R.C. performed data analysis. Y.-T.H., H.R.C. and T.H.G. wrote, reviewed, and edited the original draft. All authors have read, reviewed, and approved the final version of this paper.

DECLARATION OF INTERESTS

Commercial funding for this study was provided by NeuroGene (to T.H.G). T.H.G. has served on advisory boards for Roche, Novartis, LifeArc, and SMA Europe. R.D.H. and S.R.C. are consultants at Neurogene Inc.

REFERENCES

1. Li, L., Xiong, W.-C., and Mei, L. (2018). Neuromuscular Junction Formation, Aging, and Disorders. Annu. Rev. Physiol. *80*, 159–188. https://doi.org/10.1146/annurev-physiol-022516-034255.
2. Jones, R.A., Harrison, C., Eaton, S.L., Llavero Hurtado, M., Graham, L.C., Alkhammash, L., Oladiran, O.A., Gale, A., Lamont, D.J., Simpson, H., et al. (2017). Cellular and Molecular Anatomy of the Human Neuromuscular Junction. Cell Rep. *21*, 2348–2356. https://doi.org/10.1016/j.celrep.2017.11.008.
3. Sanes, J.R., and Lichtman, J.W. (1999). Development of the Vertebrate Neuromuscular Junction. Annu. Rev. Neurosci. *22*, 389–442. https://doi.org/10.1146/annurev.neuro.22.1.389.
4. Nishimune, H., and Shigemoto, K. (2018). Practical anatomy of the neuromuscular junction in health and disease. Neurol. Clin. *36*, 231–240. https://doi.org/10.1016/j.ncl.2018.01.009.
5. Sirago, G., Pellegrino, M.A., Bottinelli, R., Franchi, M.V., and Narici, M.V. (2023). Loss of neuromuscular junction integrity and muscle atrophy in skeletal muscle disuse. Ageing Res. Rev. *83*, 101810. https://doi.org/10.1016/j.arr.2022.101810.
6. Alhindi, A., Boehm, I., and Chaytow, H. (2022). Small junction, big problems: Neuromuscular junction pathology in mouse models of amyotrophic lateral sclerosis (ALS). J. Anat. *241*, 1089–1107. https://doi.org/10.1111/joa.13463.
7. Yang, X., Arber, S., William, C., Li, L., Tanabe, Y., Jessell, T.M., Birchmeier, C., and Burden, S.J. (2001). Patterning of muscle acetylcholine receptor gene expression in the absence of motor innervation. Neuron *30*, 399–410. https://doi.org/10.1016/s0896-6273(01)00287-2.
8. DeChiara, T.M., Bowen, D.C., Valenzuela, D.M., Simmons, M.V., Poueymirou, W.T., Thomas, S., Kinetz, E., Compton, D.L., Rojas, E., Park, J.S., et al. (1996). The Receptor Tyrosine Kinase MuSK Is Required for Neuromuscular Junction Formation In Vivo. Cell *85*, 501–512. https://doi.org/10.1016/S0092-8674(00)81251-9.
9. Hesser, B.A., Henschel, O., and Witzemann, V. (2006). Synapse disassembly and formation of new synapses in postnatal muscle upon conditional inactivation of MuSK. Mol. Cell. Neurosci. *31*, 470–480. https://doi.org/10.1016/j.mcn.2005.10.020.
10. Kong, X.C., Barzaghi, P., and Ruegg, M.A. (2004). Inhibition of synapse assembly in mammalian muscle *in vivo* by RNA interference. EMBO Rep. *5*, 183–188. https://doi.org/10.1038/sj.embor.7400065.
11. Herbst, R., Avetisova, E., and Burden, S.J. (2002). Restoration of synapse formation in Musk mutant mice expressing a Musk/Trk chimeric receptor. Development *129*, 5449–5460. https://doi.org/10.1242/dev.00112.
12. Okada, K., Inoue, A., Okada, M., Murata, Y., Kakuta, S., Jigami, T., Kubo, S., Shiraishi, H., Eguchi, K., Motomura, M., et al. (2006). The Muscle Protein Dok-7 Is Essential for Neuromuscular Synaptogenesis. Science *312*, 1802–1805. https://doi.org/10.1126/science.1127142.
13. Bergamin, E., Hallock, P.T., Burden, S.J., and Hubbard, S.R. (2010). The cytoplasmic adaptor protein Dok7 activates the receptor tyrosine kinase MuSK via dimerization. Mol. Cell. *39*, 100–109. https://doi.org/10.1016/j.molcel.2010.06.007.
14. Carpino, N., Wisniewski, D., Strife, A., Marshak, D., Kobayashi, R., Stillman, B., and Clarkson, B. (1997). p62dok: A Constitutively Tyrosine-Phosphorylated, GAP-Associated Protein in Chronic Myelogenous Leukemia Progenitor Cells. Cell *88*, 197–204. https://doi.org/10.1016/S0092-8674(00)81840-1.
15. Yamanashi, Y., and Baltimore, D. (1997). Identification of the Abl- and rasGAP-Associated 62 kDa Protein as a Docking Protein, Dok. Cell *88*, 205–211. https://doi.org/10.1016/S0092-8674(00)81841-3.
16. Beeson, D., Higuchi, O., Palace, J., Cossins, J., Spearman, H., Maxwell, S., Newsom-Davis, J., Burke, G., Fawcett, P., Motomura, M., et al. (2006). Dok-7 Mutations Underlie a Neuromuscular Junction Synaptopathy. Science *313*, 1975–1978. https://doi.org/10.1126/science.1130837.
17. Selcen, D., Milone, M., Shen, X.-M., Harper, C.M., Stans, A.A., Wieben, E.D., and Engel, A.G. (2008). Dok-7 Myasthenia: Phenotypic and Molecular Genetic Studies in 16 Patients. Ann. Neurol. *64*, 71–87. https://doi.org/10.1002/ana.21408.
18. Cossins, J., Liu, W.W., Belaya, K., Maxwell, S., Oldridge, M., Lester, T., Robb, S., and Beeson, D. (2012). The spectrum of mutations that underlie the neuromuscular junction synaptopathy in DOK7 congenital myasthenic syndrome. Hum. Mol. Genet. *21*, 3765–3775. https://doi.org/10.1093/hmg/dds198.
19. Ueta, R., Tezuka, T., Izawa, Y., Miyoshi, S., Nagatoishi, S., Tsumoto, K., and Yamanashi, Y. (2017). The carboxyl-terminal region of Dok-7 plays a key, but not essential, role in activation of muscle-specific receptor kinase MuSK and neuromuscular synapse formation. J. Biochem. *161*, 269–277. https://doi.org/10.1093/jb/mvw073.
20. Hamuro, J., Higuchi, O., Okada, K., Ueno, M., Iemura, S.I., Natsume, T., Spearman, H., Beeson, D., and Yamanashi, Y. (2008). Mutations Causing DOK7 Congenital Myasthenia Ablate Functional Motifs in Dok-7. J. Biol. Chem. *283*, 5518–5524. https://doi.org/10.1074/jbc.M708607200.
21. Oury, J., Zhang, W., Leloup, N., Koide, A., Corrado, A.D., Ketavarapu, G., Hattori, T., Koide, S., and Burden, S.J. (2021). Mechanism of disease and therapeutic rescue of Dok7 congenital myasthenia. Nature *595*, 404–408. https://doi.org/10.1038/s41586-021-03672-3.
22. Arimura, S., Okada, T., Tezuka, T., Chiyo, T., Kasahara, Y., Yoshimura, T., Motomura, M., Yoshida, N., Beeson, D., Takeda, S., and Yamanashi, Y. (2014). DOK7 gene therapy benefits mouse models of diseases characterized by defects in the neuromuscular junction. Science *345*, 1505–1508. https://doi.org/10.1126/science.1250744.
23. Ueta, R., Sugita, S., Minegishi, Y., Shimotoyodome, A., Ota, N., Ogiso, N., Eguchi, T., and Yamanashi, Y. (2020). DOK7 Gene Therapy Enhances Neuromuscular Junction Innervation and Motor Function in Aged Mice. iScience *23*, 101385. https://doi.org/10.1016/j.isci.2020.101385.
24. Bao, Z., Cui, C., Chow, S.K.-H., Qin, L., Wong, R.M.Y., and Cheung, W.-H. (2020). AChRs Degeneration at NMJ in Aging-Associated Sarcopenia–A Systematic Review. Front. Aging Neurosci. *12*, 597811. https://doi.org/10.3389/fnagi.2020.597811.
25. Woschitz, V., Mei, I., Hedlund, E., and Murray, L.M. (2022). Mouse models of SMA show divergent patterns of neuronal vulnerability and resilience. Skeletal Muscle *12*, 22. https://doi.org/10.1186/s13395-022-00305-9.
26. Murray, L.M., Comley, L.H., Thomson, D., Parkinson, N., Talbot, K., and Gillingwater, T.H. (2008). Selective vulnerability of motor neurons and dissociation of pre- and post-synaptic pathology at the neuromuscular junction in mouse models of spinal muscular atrophy. Hum. Mol. Genet. *17*, 949–962. https://doi.org/10.1093/hmg/ddm367.
27. Mejia Maza, A., Jarvis, S., Lee, W.C., Cunningham, T.J., Schiavo, G., Secrier, M., Fratta, P., Sleigh, J.N., Fisher, E.M.C., and Sudre, C.H. (2021). NMJ-Analyser identifies subtle early changes in mouse models of neuromuscular disease. Sci. Rep. *11*, 12251. https://doi.org/10.1038/s41598-021-91094-6.
28. Rodríguez Cruz, P.M., Cossins, J., Beeson, D., and Vincent, A. (2020). The Neuromuscular Junction in Health and Disease: Molecular Mechanisms Governing Synaptic Formation and Homeostasis. Front. Mol. Neurosci. *13*, 610964. https://doi.org/10.3389/fnmol.2020.610964.
29. Miyoshi, S., Tezuka, T., Arimura, S., Tomono, T., Okada, T., and Yamanashi, Y. (2017). DOK7 gene therapy enhances motor activity and life span in ALS model mice. EMBO Mol. Med. *9*, 880–889. https://doi.org/10.15252/emmm.201607298.
30. Kaifer, K.A., Villalón, E., Smith, C.E., Simon, M.E., Marquez, J., Hopkins, A.E., Morcos, T.I., and Lorson, C.L. (2020). AAV9-DOK7 gene therapy reduces disease severity in Smn2B/- SMA model mice. Biochem. Biophys. Res. Commun. *530*, 107–114. https://doi.org/10.1016/j.bbrc.2020.07.031.
31. Kosco, E.D., Jing, H., Chen, P., Xiong, W.-C., Samuels, I.S., and Mei, L. (2023). DOK7 Promotes NMJ Regeneration After Nerve Injury. Mol. Neurobiol. *60*, 1453–1464. https://doi.org/10.1007/s12035-022-03143-4.
32. White, R.B., Biérinx, A.S., Gnocchi, V.F., and Zammit, P.S. (2010). Dynamics of muscle fibre growth during postnatal mouse development. BMC Dev. Biol. *10*, 21. https://doi.org/10.1186/1471-213X-10-21.
33. Jones, R.A., Reich, C.D., Dissanayake, K.N., Kristmundsdottir, F., Findlater, G.S., Ribchester, R.R., Simmen, M.W., and Gillingwater, T.H. (2016). NMJ-morph reveals principal components of synaptic morphology influencing structure–function relationships at the neuromuscular junction. Open Biol. *6*, 160240. https://doi.org/10.1098/rsob.160240.
34. Boehm, I., Miller, J., Wishart, T.M., Wigmore, S.J., Skipworth, R.J., Jones, R.A., and Gillingwater, T.H. (2020). Neuromuscular junctions are stable in patients with cancer cachexia. J. Clin. Invest. *130*, 1461–1465. https://doi.org/10.1172/JCI128411.

35. Miller, T.M., Cudkowicz, M.E., Genge, A., Shaw, P.J., Sobue, G., Bucelli, R.C., Chiò, A., Van Damme, P., Ludolph, A.C., Glass, J.D., et al. (2022). Trial of Antisense Oligonucleotide Tofersen for SOD1 ALS. N. Engl. J. Med. *387*, 1099–1110. https://doi.org/10.1056/NEJMoa2204705.

36. Mendell, J.R., Al-Zaidy, S., Shell, R., Arnold, W.D., Rodino-Klapac, L.R., Prior, T.W., Lowes, L., Alfano, L., Berry, K., Church, K., et al. (2017). Single-Dose Gene-Replacement Therapy for Spinal Muscular Atrophy. N. Engl. J. Med. *377*, 1713–1722. https://doi.org/10.1056/NEJMoa1706198.

37. Zincarelli, C., Soltys, S., Rengo, G., and Rabinowitz, J.E. (2008). Analysis of AAV serotypes 1-9 mediated gene expression and tropism in mice after systemic injection. Mol. Ther. *16*, 1073–1080. https://doi.org/10.1038/mt.2008.76.

38. Webster, R.G., Vanhaesebrouck, A.E., Maxwell, S.E., Cossins, J.A., Liu, W., Ueta, R., Yamanashi, Y., and Beeson, D.M.W. (2020). Effect of salbutamol on neuromuscular junction function and structure in a mouse model of DOK7 congenital myasthenia. Hum. Mol. Genet. *29*, 2325–2336. https://doi.org/10.1093/hmg/ddaa116.

39. Hammond, S.M., Gogliotti, R.G., Rao, V., Beauvais, A., Kothary, R., and DiDonato, C.J. (2010). Mouse survival motor neuron alleles that mimic SMN2 splicing and are inducible rescue embryonic lethality early in development but not late. PLoS One *5*, e15887. https://doi.org/10.1371/journal.pone.0015887.

40. Gordon, D., Dafinca, R., Scaber, J., Alegre-Abarrategui, J., Farrimond, L., Scott, C., Biggs, D., Kent, L., Oliver, P.L., Davies, B., et al. (2019). Single-copy expression of an amyotrophic lateral sclerosis-linked TDP-43 mutation (M337V) in BAC transgenic mice leads to altered stress granule dynamics and progressive motor dysfunction. Neurobiol. Dis. *121*, 148–162. https://doi.org/10.1016/j.nbd.2018.09.024.

41. Gaist, D., Mogensen, J., Pedersen, E.G., Schrøder, H.D., Vissing, J., Andersen, H., and Hertz, J.M. (2017). DOK7 congenital myasthenia may be associated with severe mitral valve insufficiency. J. Neurol. Sci. *379*, 217–218. https://doi.org/10.1016/j.jns.2017.06.005.

42. Valenzuela, D.M., Stitt, T.N., DiStefano, P.S., Rojas, E., Mattsson, K., Compton, D.L., Nuñez, L., Park, J.S., Stark, J.L., Gies, D.R., et al. (1995). Receptor tyrosine kinase specific for the skeletal muscle lineage: Expression in embryonic muscle, at the neuromuscular junction, and after injury. Neuron *15*, 573–584. https://doi.org/10.1016/0896-6273(95)90146-9.

43. Powis, R.A., Karyka, E., Boyd, P., Côme, J., Jones, R.A., Zheng, Y., Szunyogova, E., Groen, E.J., Hunter, G., Thomson, D., et al. (2016). Systemic restoration of UBA1 ameliorates disease in spinal muscular atrophy. JCI Insight *1*, e87908. https://doi.org/10.1172/jci.insight.87908.

44. Huang, Y.-T., van der Hoorn, D., Ledahawsky, L.M., Motyl, A.A.L., Jordan, C.Y., Gillingwater, T.H., and Groen, E.J.N. (2019). Robust Comparison of Protein Levels Across Tissues and Throughout Development Using Standardized Quantitative Western Blotting. J. Vis. Exp. https://doi.org/10.3791/59438.

45. Groen, E.J.N., Perenthaler, E., Courtney, N.L., Jordan, C.Y., Shorrock, H.K., van der Hoorn, D., Huang, Y.-T., Murray, L.M., Viero, G., and Gillingwater, T.H. (2018). Temporal and tissue-specific variability of SMN protein levels in mouse models of spinal muscular atrophy. Hum. Mol. Genet. *27*, 2851–2862. https://doi.org/10.1093/hmg/ddy195.

46. Eaton, S.L., Roche, S.L., Llavero Hurtado, M., Oldknow, K.J., Farquharson, C., Gillingwater, T.H., and Wishart, T.M. (2013). Total Protein Analysis as a Reliable Loading Control for Quantitative Fluorescent Western Blotting. PLoS One *8*, e72457. https://doi.org/10.1071/journal.pone.0072457.

47. Zhao, K., Shen, C., Li, L., Wu, H., Xing, G., Dong, Z., Jing, H., Chen, W., Zhang, H., Tan, Z., et al. (2018). Sarcoglycan Alpha Mitigates Neuromuscular Junction Decline in Aged Mice by Stabilizing LRP4. J. Neurosci. *38*, 8860–8873. https://doi.org/10.1523/JNEUROSCI.0860-18.2018.

48. Zhang, S., Ohkawara, B., Ito, M., Huang, Z., Zhao, F., Nakata, T., Takeuchi, T., Sakurai, H., Komaki, H., Kamon, M., et al. (2023). A mutation in DOK7 in congenital myasthenic syndrome forms aggresome in cultured cells, and reduces DOK7 expression and MuSK phosphorylation in patient-derived iPS cells. Hum. Mol. Genet. *32*, 1511–1523. https://doi.org/10.1093/hmg/ddac306.

OMTN, Volume *33*

Supplemental information

Long-term muscle-specific overexpression of DOK7 in mice using AAV9-tMCK-DOK7

Yu-Ting Huang, Hannah R. Crick, Helena Chaytow, Dinja van der Hoorn, Abrar Alhindi, Ross A. Jones, Ralph D. Hector, Stuart R. Cobb, and Thomas H. Gillingwater

Table S1. Histopathology incidence in 3-month-old animals

Tissue	Sex		Low Dose	High Dose	Untreated	Finding incidence	Finding Remark
Brain	M	No Examined	1	1	1	-	
		No. Unremarkable	1	1	1	0	
	F	No Examined	2	2	2	-	
		No. Unremarkable	2	2	2	0	
Diaphragm	M	No Examined	1	1	1	-	
		No. Unremarkable	1	1	1	0	
	F	No Examined	2	2	2	-	
		No. Unremarkable	2	2	2	0	
Heart	M	No Examined	1	1	1	-	
		No. Unremarkable	1	1	1	0	
	F	No Examined	2	2	2	-	
		No. Unremarkable	2	2	2	0	
Kidney	M	No Examined	1	1	1	-	
		No. Unremarkable	1	1	1	0	
	F	No Examined	2	2	2	-	
		No. Unremarkable	1	2	2	1	Hypertrophy
Liver	M	No Examined	1	1	1	-	
		No. Unremarkable	1	1	0	1	Mixed cell infiltration
	F	No Examined	2	2	2	-	
		No. Unremarkable	2	1	0	3	Mixed cell infiltration
Lungs	M	No Examined	1	1	1	-	
		No. Unremarkable	1	1	1	0	
	F	No Examined	2	2	2	-	
		No. Unremarkable	2	2	2	0	
Soleus	M	No Examined	1	1	1	-	
		No. Unremarkable	1	1	1	0	
	F	No Examined	2	2	2	-	
		No. Unremarkable	2	2	2	0	
Spleen	M	No Examined	1	1	1	-	

		No. Unremarkable	0	0	0	3	Extramedullary Hematopoiesis
	F	No Examined	2	2	2	-	
		No. Unremarkable	0	0	0	3	Extramedullary Hematopoiesis
Testes	M	No Examined	1	1	1	-	
		No. Unremarkable	1	1	1	0	

Table S2. Histopathology incidence in 6-month animals

Tissue	Sex		Low Dose	High Dose	Untreated	Finding incidence	Finding Remark
Brain	M	No Examined	2	2	2	-	
		No. Unremarkable	2	2	2	0	
	F	No Examined	1	1	1	-	
		No. Unremarkable	1	1	1	0	
Diaphragm	M	No Examined	2	2	2	-	
		No. Unremarkable	2	1	2	1	Myofibre degeneration
	F	No Examined	1	1	1	-	
		No. Unremarkable	1	1	1	0	
Heart	M	No Examined	2	2	2	-	
		No. Unremarkable	2	2	2	0	
	F	No Examined	1	1	1	-	
		No. Unremarkable	1	0	1	1	Mononuclear cell infiltration
Kidney	M	No Examined	2	2	2	-	
		No. Unremarkable	1	2	2	1	Tubular basophilia
	F	No Examined	1	1	1	-	
		No. Unremarkable	1	1	1	0	
Liver	M	No Examined	2	2	2	-	
		No. Unremarkable	1	1	2	2	Mixed cell infiltration
	F	No Examined	1	1	1	-	
		No. Unremarkable	0	1	0	2	Mixed cell infiltration
Lungs	M	No Examined	2	2	2	-	
		No. Unremarkable	2	2	2	0	
	F	No Examined	1	1	1	-	

		No. Unremarkable	1	1	1	0	
Soleus	M	No Examined	2	2	2	-	
		No. Unremarkable	2	0	2	2	Myofibre degeneration
	F	No Examined	1	1	1	-	
		No. Unremarkable	0	1	0	2	Myofibre degeneration
			0	0	1	2	Myofibre regeneration
Spleen	M	No Examined	2	2	2	-	
		No. Unremarkable	0	0	0	6	Extramedullary Hematopoiesis
	F	No Examined	1	1	1	-	
		No. Unremarkable	0	0	0	3	Extramedullary Hematopoiesis
Testes	M	No Examined	2	2	2	-	
		No. Unremarkable	2	2	2	0	
Ovary	F	No Examined	1	1	1	-	
		No. Unremarkable	1	1	1	0	

Table S3. Incidence and severity of select Jasmone-related microscopic findings in the soleus muscle at 6 months

		Males			Females		
	Treatment	Untreated	Low Dose	High Dose	Untreated	Low Dose	High Dose
	No Animals	3	3	3	3	3	3
Myofibre Degeneration	Minimal	0	0	2	0	1	0
	Mild	0	0	0	1	0	0
Myofibre Regeneration	Minimal	0	0	0	0	1	1

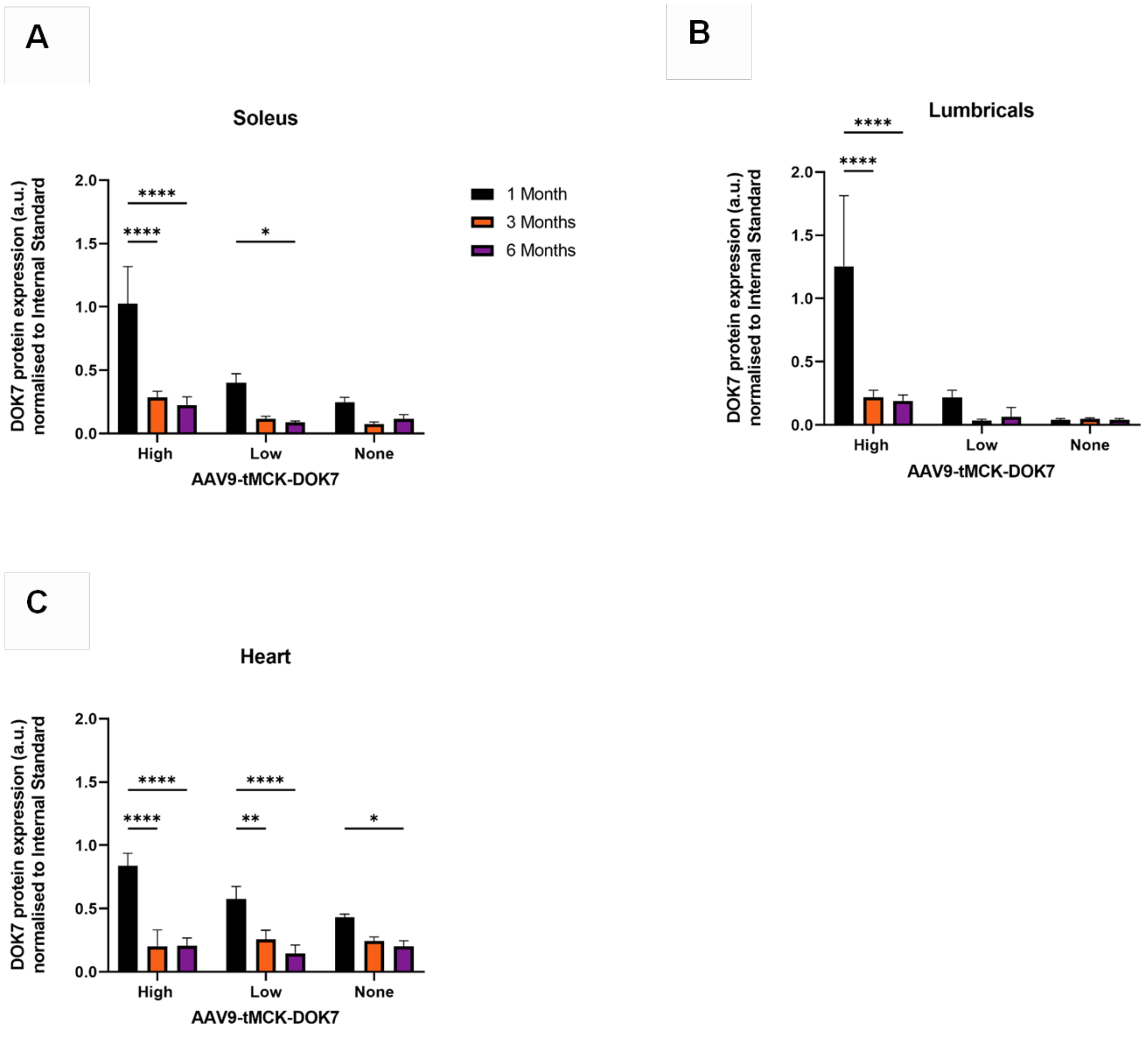

Figure S1. Changes in DOK7 expression over 6 months following AAV9-tMCK-DOK7 treatment

(A) In the soleus muscle DOK7 expression significantly decreased between 1 month of age/post-injection and 3 months following high-dose treatment, and between 1 and 6 months following high and low dose treatments. (B) In the hindlimb lumbrical muscles DOK7 protein expression was significantly decreased after 1-month following injection of the hihg dose only. (C) There was a decrease in endogenous DOK7 levels between 1 and 6-months of age in the heart in untreated animals, as well as a decrease following high and low dose treatment. Data are presented as means +/- SEM. One-way ANOVAs with Sidak's multiple comparisons. **** $p < 0.0001$, **$p < 0.01$, *$p < 0.05$.

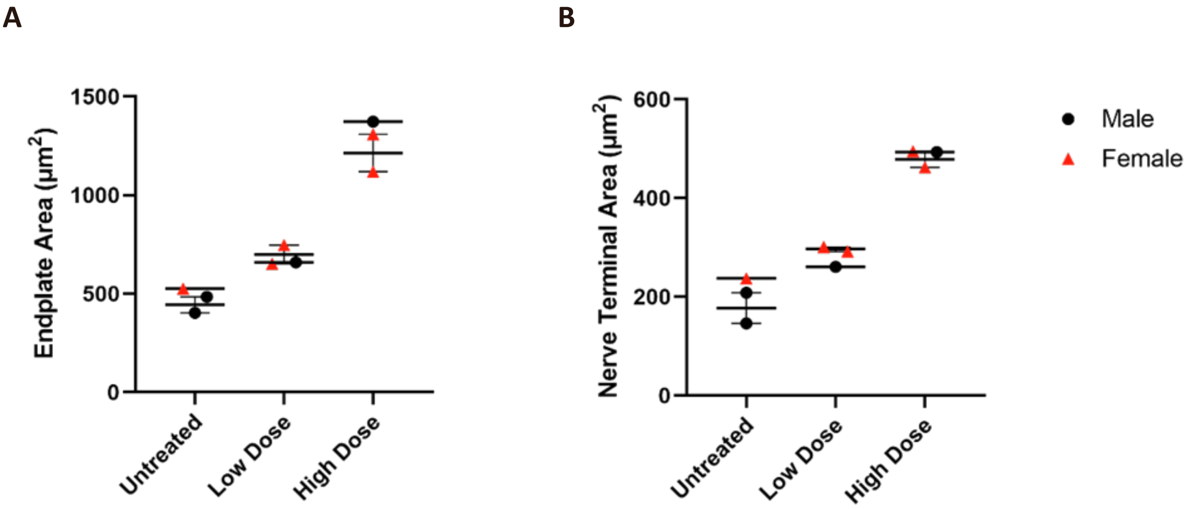

Figure S2. Sex does not impact NMJ parameters in 6-month lumbrical muscles.

Data points produced from female mice are highlighted as red triangles, and those from male mice in black circles. In endplate area (A) and nerve terminal area (B) there is no evidence that sex affects NMJ size in response to AAV9-tMCK-DOK7 treatment at a low or high dose, nor a natural distinction between untreated male and female mice.
